# The Perceived Roles of AI in Clinical Practice: National Survey of 941 Academic Physicians

**DOI:** 10.2196/72535

**Published:** 2025-12-04

**Authors:** Anshul Ratnaparkhi, Simon Moore, Abhinav Suri, Bayard Wilson, Jacob Alderete, TJ Florence, David Zarrin, David Berin, Rami Abuqubo, Kirstin Cook, Matiar Jafari, Joseph S Bell, Luke Macyszyn, Andrew C Vivas, Joel Beckett

**Affiliations:** 1 Department of Neurosurgery David Geffen School of Medicine University of California, Los Angeles Los Angeles, CA United States; 2 David Geffen School of Medicine University of California, Los Angeles Los Angeles, CA United States

**Keywords:** artificial intelligence in medicine, machine learning in health care, clinical decision support systems, AI adoption, medical informatics, physician attitudes, barriers to AI adoption, health care technology acceptance

## Abstract

**Background:**

Artificial intelligence (AI) and machine learning models are frequently developed in medical research to optimize patient care, yet they remain rarely used in clinical practice.

**Objective:**

This study aims to understand the disconnect between model development and implementation by surveying physicians of all specialties across the United States.

**Methods:**

The present survey was distributed to residency coordinators at Accreditation Council for Graduate Medical Education–accredited residency programs to disseminate among attending physicians and resident physicians affiliated with their academic institution. Respondents were asked to identify and quantify the extent of their training and specialization, as well as the type and location of their practice. Physicians were then asked follow-up questions regarding AI in their practice, including whether its use is permitted, whether they would use it if made available, primary reasons for using or not using AI, elements that would encourage its use, and ethical concerns.

**Results:**

Of the 941 physicians who responded to the survey, 384 (40.8%) were attending physicians and 557 (59.2%) were resident physicians. The majority of the physicians (651/795, 81.9%) indicated that they would adopt AI in clinical practice if given the opportunity. The most cited intended uses for AI were risk stratification, image analysis or segmentation, and disease prognosis. The most common reservations were concerns about clinical errors made by AI and the potential to replicate human biases.

**Conclusions:**

To date, this study comprises the largest and most diverse dataset of physician perspectives on AI. Our results emphasize that most academic physicians in the United States are open to adopting AI in their clinical practice. However, for AI to become clinically relevant, developers and physicians must work synergistically to design models that are accurate, accessible, and intuitive while thoroughly addressing ethical concerns associated with the implementation of AI in medicine.

## Introduction

### Background

Artificial intelligence (AI) is a topic of significant interest in recent literature. It has garnered considerable attention as a potential tool to aid in detecting, diagnosing, and managing diseases and demonstrated efficacy in detecting diabetic retinopathy, diagnosing skin cancers, and even predicting patients’ surgical candidacy [1-5]. As artificial intelligence grows increasingly capable, one might expect an associated increase in the clinical uses of such programs; however, AI models are rarely harnessed in medicine today outside the realm of research [6,7].

The disconnect between model development and deployment has been examined previously [8-10]. The lack of clinical implementation of AI and machine learning techniques often stems from a lack of physician trust in model accuracy and the difficulties associated with integrating models into existing clinical paradigms, such as electronic health record platforms [11]. Current articles on the topic are often authored by field experts and frequently present subjective or anecdotal findings rather than a systematic exploration of sentiments toward AI in medicine at large. For AI to realize its potential and become a clinically relevant tool, it is essential to understand how physicians foresee its use and integration.

### Objectives

This study aims to quantify key factors relevant to integrating AI into clinical medicine, including physician adoption and use, incentivization strategies, and ethical considerations. To achieve this, we developed and distributed an anonymous survey using the Qualtrics platform (Qualtrics International Inc, Provo, UT) to resident and attending physicians associated with academic institutions across all medical specialties throughout the United States. The diversity and volume of responses to this survey yielded, to our knowledge, the largest and most comprehensive dataset to date of physician perspectives on the implementation of AI. In doing this, we aim to analyze use patterns across a broad range of physicians to better understand the gap between AI model development and clinical implementation.

## Methods

### Ethical Considerations

This study was conducted according to the Declaration of Helsinki, which sets ethical principles for medical research involving human participants [12]. As the present study involved a minimal-risk, de-identified survey, formal Institutional Review Board (IRB) review was not required.

### Survey Instrument

**Figure 1:**
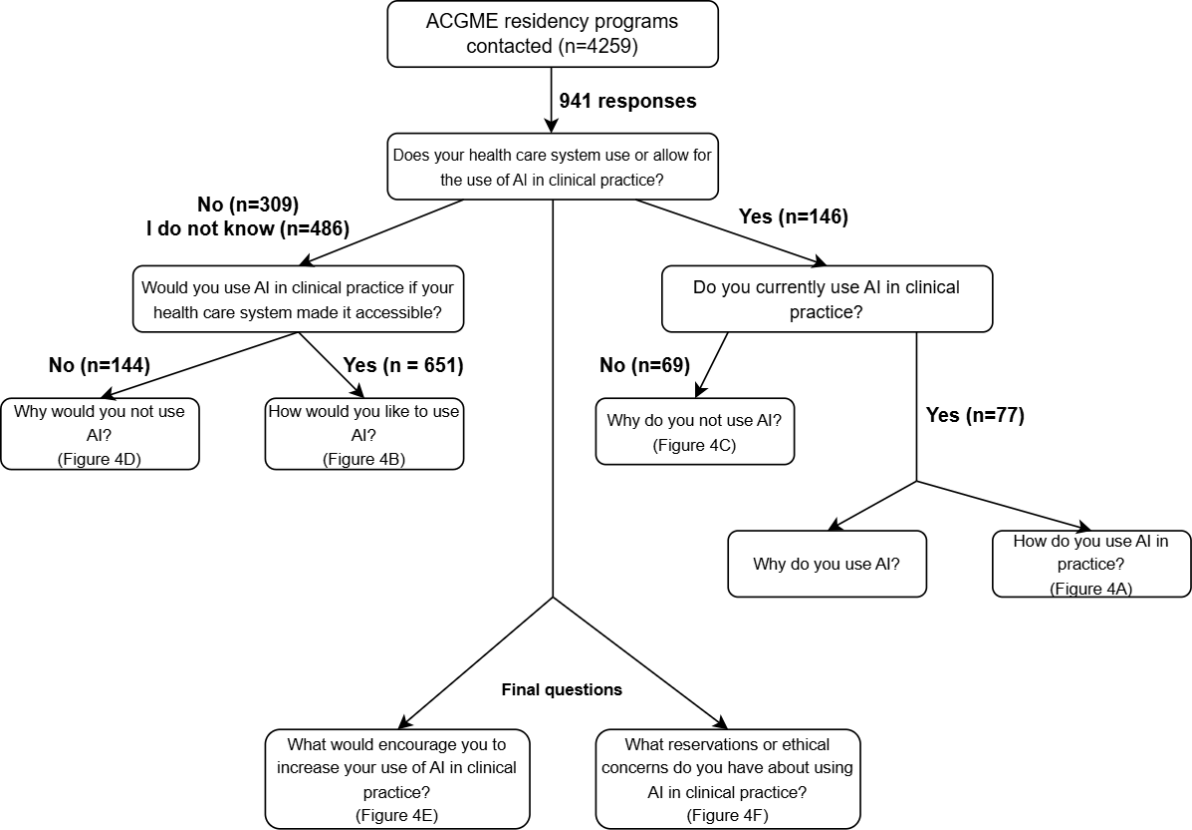
Flowchart of survey format. Participants were displayed subquestions depending on their selection of the questions shown here, presented as branches. ACGME: Accreditation Council for Graduate Medical Education; AI: artificial intelligence.

The survey was initially conceptualized and developed by 2 authors. It was subsequently reviewed independently by 5 senior coauthors; after a group discussion, relevant modifications were incorporated, and all contributing authors approved the final version of the survey. The survey was piloted at our home institution before being distributed nationally. The final 14-item survey included 7 demographic questions (eg, training status, specialty, years in practice, and practice setting), followed by 7 AI-related questions assessing awareness, intent to use, perceived use cases, incentives, and ethical concerns. Conditional branching logic was used to display specific follow-up questions based on prior responses (Figure 1) to obtain a deeper understanding of the rationale behind response selections. The complete survey is provided in Multimedia Appendix 1. The survey was designed and distributed in accordance with the CHERRIES (Checklist for Reporting Results of Internet E-Surveys) checklist [13].

### Study Design and Distribution

The survey was distributed to residency program coordinators representing 18 medical and surgical specialties in the United States: anesthesiology, dermatology, emergency medicine, family medicine, general surgery, internal medicine, neurology, neurosurgery, obstetrics and gynecology, ophthalmology, orthopedic surgery, otolaryngology, pediatrics, plastic surgery, psychiatry, radiation oncology, diagnostic radiology, and urology. These specialties were chosen to represent a diverse cross-section of core disciplines in US residency training.

This open survey used a nonprobability referral sampling design, relying on program coordinators from Accreditation Council for Graduate Medical Education (ACGME)–accredited residency programs to distribute it to associated resident physicians and attending physicians. Program coordinators’ contact information was manually obtained from the publicly accessible ACGME program search website. If an email address was invalid, the residency program’s website was queried to obtain an updated email address. Correspondence containing the Qualtrics survey was first sent to program coordinators on January 11, 2023, by 1 author. The same author sent follow-up correspondence at 1-month and 2-month intervals after the initial outreach. Program coordinators who confirmed survey distribution were excluded from follow-up communications. The survey remained open for responses until November 3, 2023, allowing for an 11-month data collection period.

Survey participation was voluntary, anonymous, and without incentives. However, cookies, approximate location, and IP address were automatically collected using the Qualtrics platform to identify and remove duplicate submissions. Only complete responses were included in this analysis. As surveys were distributed to program coordinators, who subsequently disseminated them, the true survey response rate was not documented.

### Statistical Analysis

Statistical analyses were conducted using the *SciPy* (version 1.11.4) and *Statsmodels* Python libraries. For group comparisons, chi-square tests of independence were used to evaluate differences in categorical variables. Phi coefficients were calculated as a measure of effect size for 2x2 contingency tables, and Cramér V was reported for larger contingency tables. A *P* value of <.05 was considered statistically significant. For proportion comparisons, 95% CIs were calculated using the Wilson score method. All tests were 2-tailed and conducted on complete-case data.

## Results

### Survey Respondents and Demographics

**Figure 2:**
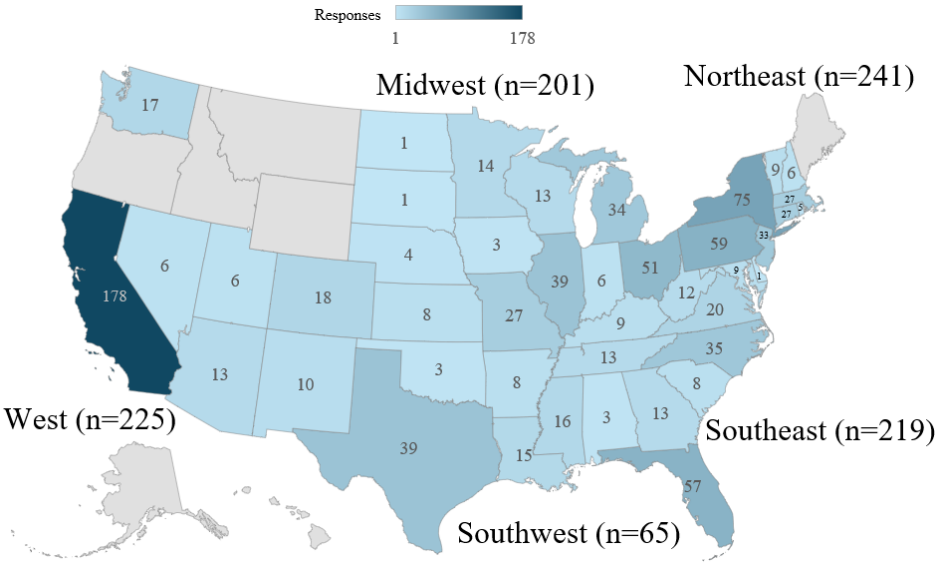
Geographic heat map of locations where physician survey participants practiced. Geographic regions were divided based on National Geographic Education outline maps. Physician submissions from California represented the largest group (n=178), while those from Delaware, Nebraska, North Dakota, and South Dakota represented the smallest group (n=1 each). Other responses not shown on the heat map include Washington DC (n=4). No responses were recorded from Alaska, Hawaii, Idaho, Maine, Montana, Oregon, or Wyoming.

Of the 4405 programs contacted, coordinators from 645 programs (14.6%) confirmed by email that they had distributed the survey. Figure 1 summarizes the number of responses to the questions and responses within conditional branch points. Table 1 presents participant demographics. A total of 941 physicians completed the survey, including 384 (40.8%) attending physicians and 557 (59.2%) resident physicians. Among attending physicians, the mean time in practice was 15.43 (SD 11.49) years. Responses were received from 43 US states and Washington, DC. Among respondents, 23.7% (225/951) practiced in the West, 21.1% (201/951) in the Midwest, 6.8% (65/951) in the Southwest, 22.9% (219/951) in the Southeast, and 25.3% (241/951) in the Northeast (Figure 2). Of the 941 participants, 10 (1.1%) reported practicing in more than 1 state; in these instances, 1 point was assigned to each state listed. No responses were recorded from Alaska, Hawaii, Idaho, Maine, Montana, Oregon, or Wyoming. Regarding practice setting, 83.2% (768/923) practiced in an urban setting, 11.8% (109/923) in a rural setting, and 5% (46/923) in a suburban setting (Table 1). Of the 923 respondents, 22 (2.4%) used the *other* free-response option. On manual review of these responses, none could be definitively classified into the aforementioned practice-setting categories; therefore, these were excluded from setting comparisons. Concerning the type of practice, 51.8% (494/954) worked in a university practice, 7% (67/954) in a government practice, 35% (334/954) in a hospital-owned practice, and 6.2% (59/954) in a physician-owned practice (Table 1). Of the 954 respondents, 67 (7%) chose *other* and were therefore excluded from type-of-practice comparisons because they did not align with the other 4 categories.

**Table 1:** Participant demographics.

Characteristics			Participants, n (%)
**Physician status (n=941)**			
	Attending physician	384 (40.8)	
	Resident physician	557 (59.2)	
**Specialty (n=941)**			
	Anesthesiology	58 (6.2)	
	Dermatology	44 (4.7)	
	Emergency medicine	91 (9.7)	
	Family medicine	84 (8.9)	
	General surgery	73 (7.8)	
	Internal medicine	147 (15.6)	
	Neurology	22 (2.3)	
	Neurosurgery	32 (3.4)	
	Obstetrics and gynecology	64 (6.8)	
	Ophthalmology	39 (4.1)	
	Orthopedic surgery	37 (3.9)	
	Otolaryngology	37 (3.9)	
	Pediatrics	41 (4.4)	
	Plastic surgery	21 (2.2)	
	Psychiatry	80 (8.5)	
	Radiation oncology	30 (3.2)	
	Diagnostic radiology	20 (2.1)	
	Urology	21 (2.2)	
**Type of specialty (n=941)**			
	Medical^a^	617 (65.6)	
	Surgical	324 (34.4)	
**Practice setting (n=923)**			
	Urban	768 (83.2)	
	Rural	109 (11.8)	
	Suburban	46 (5)	
	Other	22 (2.4)	
**Type of practice (n=954)**			
	University	494 (51.8)	
	Government	67 (7)	
	Hospital owned	334 (35)	
	Physician owned	59 (6.2)	
	Other	67 (7)	
**Region (n=951)**			
	West	225 (23.7)	
	Midwest	201 (21.1)	
	Southwest	65 (6.8)	
	Southeast	219 (23)	
	Northeast	241 (25.3)	

^a^Anesthesiology, dermatology, emergency medicine, family medicine, internal medicine, neurology, pediatrics, psychiatry, radiation oncology, diagnostic radiology, and oncology.

### The Use of AI in Clinical Medicine

**Figure 3:**
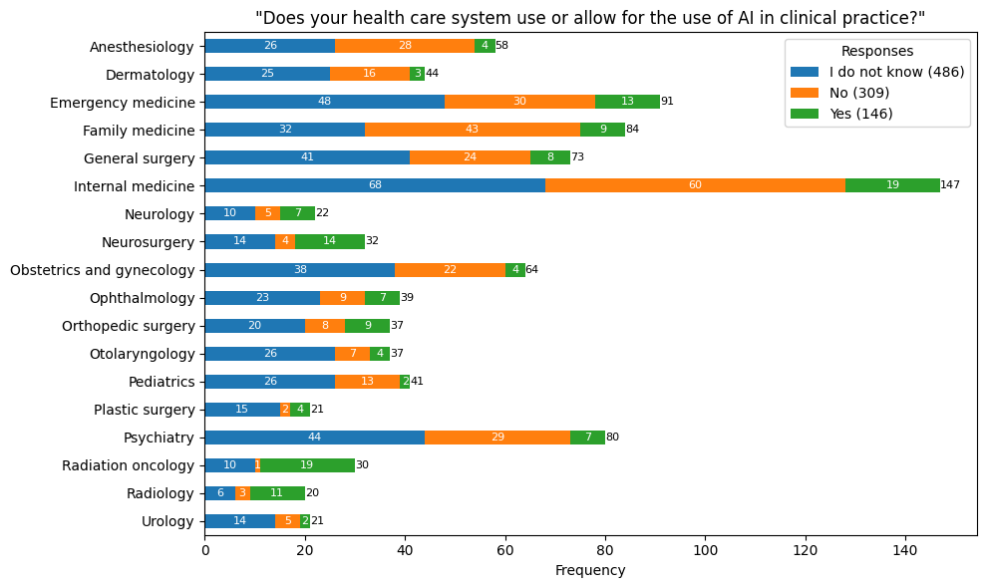
Artificial intelligence (AI) use in clinical practice by specialty. A total of 941 physicians from 18 specialties indicated whether their health care system allowed AI use in clinical practice. This was the first survey question displayed to all participants, and they were required to select only from the 3 options shown.

Most of the participants (795/941, 84.5%) were either unaware whether using AI was permissible in their practice or could not use AI in their health care setting. Figure 3 summarizes the responses to this survey question by physician specialty. Of the 795 respondents, 651 (81.9%) stated that they would use AI if their health care setting permitted it. Table 2 shows comparisons in responses to this question stratified by training status (resident physician vs attending physician), practice setting, and type of practice. Significantly more resident physicians (407/481, 84.6%, 95% CI 81.1%-87.6%) were open to using AI than attending physicians (244/314, 77.7%, 95% CI 72.8%-82%; *χ*^2^=5.7; φ=0.08; *P*=.02). Similarly, a significantly higher proportion of urban practitioners (538/646, 83.3%) were open to using AI than rural practitioners (68/94, 72.3%, 95% CI 62.6%-80.4%; *χ*^2^=5.9; φ=0.09; *P*=.03). There was no statistically significant difference in willingness to use AI between types of practice (*χ*^2^=0.2; Cramér V=0.02; *P*=.97).

**Table 2:** Willingness to adopt artificial intelligence (AI) stratified by participant characteristics.

Characteristics		Willing to adopt AI, n (%)	Not willing to adopt AI, n (%)
**Physician status**			
	Attending (n=314)	244 (77.7)	70 (22.3)
	Resident (n=481)	407 (84.6)	74 (15.4)
**Practice setting^a^**			
	Urban (n=646)	538 (83.3)	108 (16.7)
	Suburban (n=39)	31 (79.5)	8 (20.5)
	Rural (n=94)	68 (72.3)	26 (27.7)
**Type of practice^a^**			
	Government (n=61)	51 (83.6)	10 (16.4)
	Hospital owned (n= 270)	225 (83.3)	45 (16.7)
	Physician owned (n= 52)	43 (82.7)	9 (17.3)
	University (n= 412)	338 (82.0)	74 (18)

^a^Physicians were able to select multiple options if they practiced in more than 1 state.

**Figure 4:**
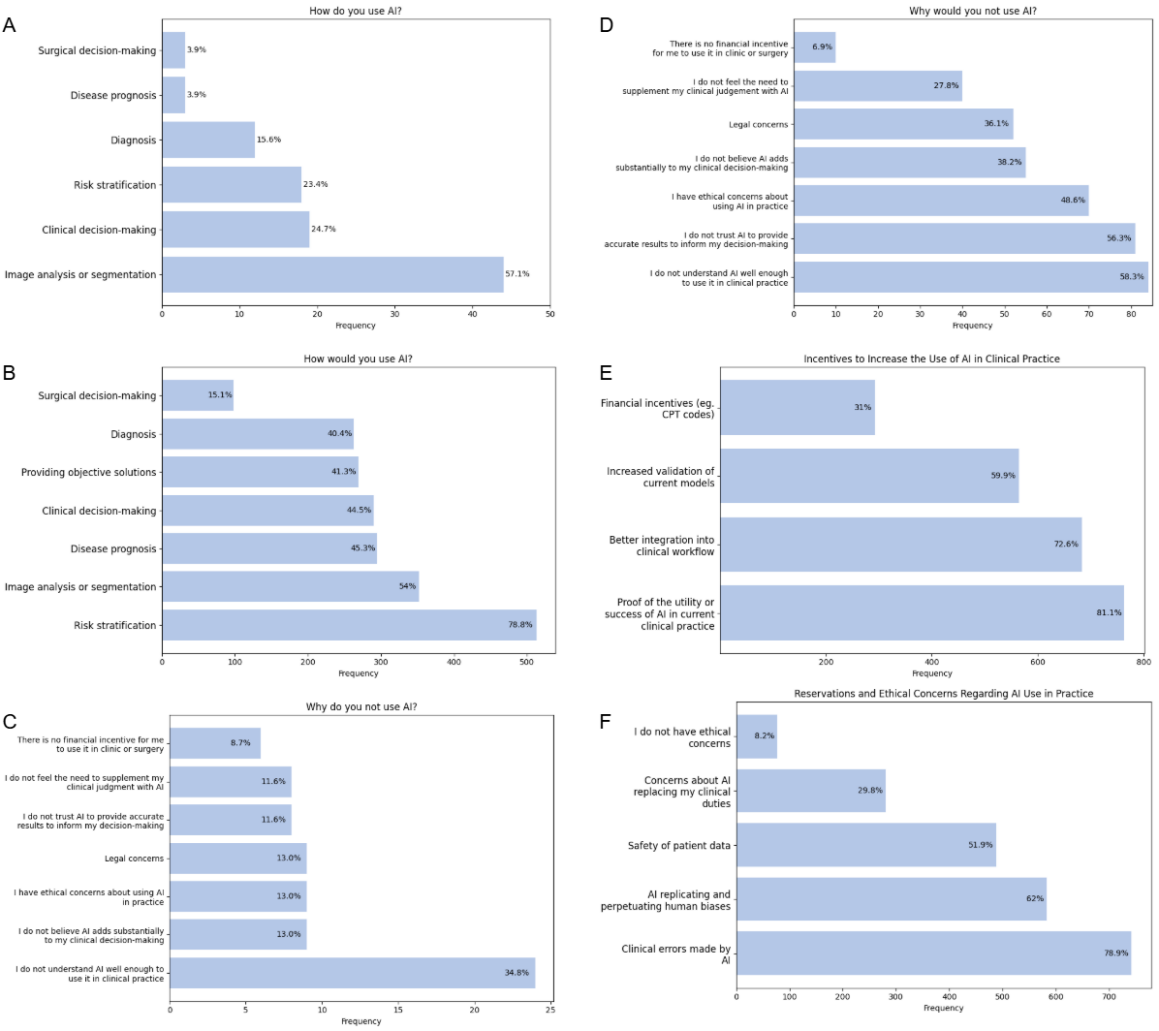
Recorded responses to survey questions. Participants were able to select multiple options. (A) Current reported uses of artificial intelligence (AI) in clinical practice (n=77). (B) Desired uses of AI if it were permitted (n=651). (C) Reported reasons given by physicians (n=69) for not using AI in clinical practice. (D) Reported reasons for not using AI if it were permitted (n=144). Physicians who were unable to use AI or who were unsure whether it was permitted in their practice and selected that they would not use AI were displayed this question (n=144). (E) Stated incentives that would increase the physicians’ use of AI in clinical practice (n=941). (F) Stated reservations and ethical concerns regarding AI use in clinical practice (n=941). CPT: current procedural terminology.

Data were also collected regarding how physicians would use AI if given the opportunity and explored factors influencing their present use or nonuse (Figure 4 A-C). Among the respondents who indicated that they would use AI if allowed, the most common applications were risk stratification (513/651,78.8%), image analysis or segmentation (352/651, 54.1%), and disease prognosis (295/651, 45.3%). When stratified by specialty, medicine respondents most often indicated interest in using AI for risk stratification (316/422, 74.9%), followed by image analysis or segmentation (197/422, 46.7%) and clinical decision-making (190/422, 45%), whereas surgical respondents more frequently selected risk stratification (197/229, 86%), followed by image analysis or segmentation (155/229, 67.7%) and disease prognosis (124/229, 54.1%) (Table 3). Although surgical decision-making was the least frequently selected use of AI overall (98/651, 15.1%), surgeons (66/229, 28.8%, 95% CI 23.3%-35%) were significantly more likely than nonsurgeons (32/422, 7.6%, 95% CI 5.4%-10.5%; *χ*^2^=50.7; φ=0.28; *P*<.001) to indicate interest in this application.

**Table 3:** Intended uses of artificial intelligence (AI).

Intended uses of AI	Medicine, n=422, n (%)	Surgery, n=229, n (%)	Overall (n=651), n (%)
Risk stratification	316/422 (74.9)	197/229 (86)	513 (78.8)
Image analysis or segmentation	197/422 (46.7)	155/229 (67.7)	352 (54.1)
Disease prognosis	171/422 (40.5)	124/229 (54.1)	295 (45.3)
Clinical decision-making	190/422 (45)	100/229 (43.7)	290 (44.5)
Providing objective solutions	186/422 (44.1)	83/229 (36.2)	269 (41.3)
Diagnosis	179/422 (42.4)	84/229 (36.7)	263 (40.4)
Surgical decision-making	32/422 (7.6)	66/229 (28.8)	98 (15.1)

### Opinions Regarding the Utility of and Reservations About AI

The most common incentives for wanting to use AI (Figure 4 A-C, E) were proof of utility or success of AI in clinical practice (763/941, 81.1%) and better integration into the clinical workflow (683/941, 72.6%). Among the stated reservations about using AI (Figure 4F), the most commonly listed was clinical errors made by AI (742/941, 78.9%), whereas the second most common was a concern about AI replicating and perpetuating human biases 583/941, 62%).

## Discussion

### Principal Findings

This study describes the perspectives of academic physicians on adopting AI in medicine, based on survey responses from 941 physicians across all 18 specialties accredited by the ACGME. Our results demonstrate that, broadly, physicians at academic institutions in the United States are interested in harnessing AI in their clinical practice, particularly for risk stratification, image analysis or segmentation, and disease prognosis. However, this eagerness to adopt AI is tempered by significant and pertinent ethical concerns regarding perpetuating human biases. To our knowledge, this study represents the largest and most diverse dataset to date of physician perspectives on AI.

Given the wide-ranging potential of AI, the term itself carries a high degree of ambiguity in interpretation, both in this study and society at large. [14]; for instance, the term artificial intelligence can be applied to several technologies, ranging from virtual assistants to robotic navigation systems [15]. Essentially, AI refers to the capability of an automated machine to replicate human intelligence, such as understanding and interpreting data to make decisions in ways that approximate human reasoning [16]. Our observation, that 51.6% (486/941) of the respondents were unsure about whether their health care system permitted the use of AI techniques, may be explained by this lack of a concrete definition or understanding of what the term *artificial intelligence* constitutes. Solidifying this consensus would address at least some of this observed discrepancy [17].

Despite our imperfect understanding of AI in medicine, this survey demonstrates that academic physicians are eager to adopt it in clinical practice. The majority of the physicians (651/795, 81.9%) indicated that they would use AI if given the opportunity. Among the options provided, risk stratification was cited as the most frequent intended use. Patients regularly want to know about their prognosis, how long they might live with a given disease, or their chances of experiencing a complication during a procedure [18]. The ability to input patient-specific data into an algorithm, integrate AI-derived insights, and receive individualized prognostic data would provide immense value for patients in terms of setting expectations and maintaining transparency [19].

The least frequently selected intended use of AI was surgical decision-making (98/651, 15.1%). This is partly a result of fewer surgeons comprising the cohort of respondents (324/941, 34.4%). Unsurprisingly, a significantly greater proportion of surgical respondents indicated that they might use AI to help with surgical decision-making (66/229, 28.8%; *P*<.001), but the overall proportion still remains relatively low. The results of this study suggest that surgeons foresee using AI in several areas of medicine more readily than in surgical decision-making (Table 3), including risk stratification (197/229, 86%) and image analysis or segmentation (155/229, 67.7%). This observation may in part be due to the phrase “surgical decision-making,” which could be interpreted to mean second-to-second decisions during an active procedure or surgery, in which case it might seem difficult to imagine a real-world application of AI that does not interfere with the pace or rhythm of surgery [20]. Moreover, it could be due to a lack of imagination as to what an AI model can process as input, as preoperative planning often involves synthesizing various information modalities. Without further insight into the respondents’ rationale, we can only speculate why only a few participants (98/651, 15.1%) expressed interest in using AI for surgical decision-making. Ongoing work aims to clarify why, among the options presented, surgical decision-making was perceived as the least applicable AI integration and how this perception might be addressed in future model development.

In our survey, 81.1% (763/941) of the physicians indicated that increased proof of the success of new models in clinical practice would encourage their adoption of AI techniques. This speaks to an important, whether real or perceived, drawback of AI adoption in the current health care system. Realizing the success of AI may not be sufficient for widespread adoption, as 72.6% (683/941) of that physicians felt that improving the integration between AI algorithms and clinical workflow was another important incentive for its use. This should come as no surprise; physicians are unlikely to engage with a technology that is neither trustworthy nor easy to use [21,22].

One concern about the use of AI in medicine expressed by all respondents involves the ethical considerations of using AI algorithms. Respondents seemed most concerned about the possibility of introducing nonhuman error. Such a concern is understandable but fails to appreciate the alternative status quo in which human errors abound [23]. Rather than holding AI models to the impossible standard of perfection, these models should be compared to the current paradigm in which human beings practice medicine and make errors, as the goal of AI should be to provide *improved* rather than *perfect* medical treatment [24].

Another focal concern regarding implementing AI in medical practice among respondents was the potential for replicating human biases (583/941, 62%). Several articles have cited bias as an issue that must be resolved before the clinical integration of AI [25,26]. A landmark paper by Obermeyer et al [27] found that racial bias was deeply embedded in a widely used medical algorithm for predicting health care costs. At a given risk level, Black patients were significantly sicker than White patients because the algorithm used health care costs rather than illness severity to assign risk, significantly reducing the likelihood that Black patients were identified as needing additional care. One may argue that health care expenses were—in retrospect—a poor and biased indication of health; however, such bias is well known to persist even in models trained on actual clinical decision-making trends [28,29]. This is largely due to the lack of diversity in age, racial, ethnic, and geographic distributions in the datasets used to train medical machine learning models [30,31]. As it is unknown whether a model’s prognostic accuracy applies equally—or at all—to populations that differ from the training dataset, applying models to populations that have not been validated can generate inaccurate results that mislead physicians [32]; for example, a study testing 5 most commonly used medical machine learning algorithms demonstrated significantly better performance in White patients than in other racial groups despite being trained on a large, publicly available database [33,34]. Models trained on homogeneous data—or those that fail to report the demographic data on which they were trained—are not appropriate for clinical practice, as physicians will be unable to ascertain how deploying such models in clinical practice may impact the care of the patient they are treating. This limits the application of AI in medicine and speaks to the need for both more diverse datasets and data transparency in medical machine learning. These concerns should be thoroughly addressed before widespread clinical adoption of all machine learning models.

To our knowledge, this study represents the largest and most diverse assessment to date of attitudes toward implementing AI in clinical practice. Overall, our results demonstrate that academic physicians are open to implementing AI in clinical practice, especially after specific concerns regarding model evaluation and integration are addressed. However, AI models are vulnerable to any bias present in the data on which they are trained, and thus, the success of AI in medicine will rely as much on the quality as the quantity of training data [35]. For AI to become truly beneficial, we must harness the breadth of objective patient-specific data that are becoming increasingly available, such as through wearable devices, digital phenotyping, and other objective metrics. Even for successful models, widespread adoption may be an uphill battle. We expect that as physicians become more familiar with AI and the general population grows accustomed to its use in other domains, the integration of AI into clinical practice is likely to follow.

### Comparison With Prior Work

The scope and utility of AI in medicine continue to expand alongside advances in broader society. Nevertheless, large-scale research studies exploring physician perceptions of AI in medicine are scant and focus on a narrow subset of specialties. Doraiswamy et al [36] surveyed 791 psychiatrists from 22 countries, while Pecqueux et al [37] surveyed 147 German surgeons, and De Simone et al [38] surveyed 200 members of the World Society of Emergency Surgery. To our knowledge, our sample of 941 physicians spanning 18 discrete specialties is the first of its kind in both size and scope.

### Implications for Implementation

Essential insight into strengthening the practical utility of AI may come from the two most frequent reasons cited by the minority of physicians (144/795, 18.1%) for not adopting it: (1) an insufficient understanding of AI (84/144, 58.3%) and (2) a distrust in the accuracy of AI models (81/144, 56.3%; Figure 4D). These findings suggest that AI developers still have work to do in explaining how their technology works and validating its efficacy. Some of this understanding may develop naturally over time as AI becomes more common in nonmedical settings, making it feel more familiar to physicians and the public alike. However, there are specific actions that developers could take now that might help them in this effort; for instance, the introduction of AI into medicine would benefit tremendously from explainable AI methods, which provide insight into how a model generates predictions and help counter the perception of AI as a “black box.” In addition, explainable AI methods such as gradient-weighted class activation mapping, occlusion maps, and Shapley additive explanation values should be incorporated into research papers proposing new AI use cases in medicine [39]. Ultimately, for AI to be clinically relevant, models must not only demonstrate success in addressing risk-sensitive decisions but also be intuitive and adaptable to the demands of everyday clinical practice.

### Limitations

Our study provides valuable insights into physician perspectives on AI in medicine, but several limitations should be acknowledged. First, the survey targeted only physicians affiliated with ACGME-accredited teaching institutions, which limits the generalizability of our results to the broader population of American physicians, including those in community-based settings; however, given the wide breadth of ACGME-accredited residency programs and the hospitals they serve—including many that cross-cover veterans’ and community hospitals—we believe that the diversity of respondents across specialties and geographic regions is fairly representative of academic physicians overall. Future works are needed to identify whether a similar trend exists among physicians in community-based settings. Second, the survey was designed to be distributed via residency coordinators, making it impossible to determine the number of coordinators who actually forwarded the survey compared to the number of completed responses, limiting the validity of our study. We also cannot determine, therefore, whether certain institutions, for instance, those with an interest in AI, comprised a more significant percentage of the survey responses than other institutions. It is certainly possible that institutions with an interest in AI may have made up a large portion of our response rate, thus influencing the results of our study in favor of the use of AI. Due to the anonymous nature of this study, we are unable to determine whether this bias exists. In addition, only complete responses were included in this analysis, and view, participation, and completion rates were not recorded. Finally, while our survey was designed to explore physicians’ perspectives on AI, we did not formally assess respondents’ understanding of what constitutes AI. This introduces the potential for variation in interpretation, particularly for questions about AI-related clinical applications. Future studies should include a standardized definition of AI to ensure uniform understanding among respondents.

Overall, the success of this study design depended on three key factors: (1) the availability of reliable contact information for program coordinators, (2) the coordinators’ willingness to distribute the survey, and (3) the intended respondents’ willingness to complete it. Although this study does not capture the full spectrum of American physicians and trainees, to our knowledge, it represents one of the most extensive datasets of physician perspectives on AI in medicine. We emphasize that these findings reflect the perspectives of this specific subset of respondents and are not intended to be generalized to the entire physician population.

### Conclusions

AI represents an exciting, ever-evolving domain of opportunity for improving clinical practice. However, limited understanding of AI and its utility, uncertainty regarding its performance, and concerns about the perpetuation of human biases remain critical barriers that must be overcome in the process of gaining trust among clinicians who are considering using AI in their practice. This study reflects physicians’ opinions and perceived use cases of AI across a variety of specialties and practice settings, quantifying both the reasons why they currently use or are eager to implement AI as well as the rationale for not using or preferring not to adopt AI in their practice. The most desired avenues for implementing AI in medicine were risk stratification, image analysis or segmentation, and disease prognosis, while the most cited reservations were concerns about clinical errors made by AI and the potential for replicating human biases.

Although this study does not fully represent American physicians and trainees, to our knowledge, it represents one of the largest and most diverse datasets of physician perspectives on AI in medicine. Ultimately, the data and insight provided in this work aim to lay the foundation for how AI may best be modified by scientists and harnessed by physicians to be optimally integrated into clinical practice.

## Appendix

Multimedia Appendix 1 Full survey distributed to academic attending and resident physicians.

## Abbreviations

ACGME: Accreditation Council for Graduate Medical Education
AI: artificial intelligence
CHERRIES: Checklist for Reporting Results of Internet E-Surveys

## Data Availability

The datasets generated and analyzed during this study are available from the corresponding author on reasonable request.
