# Supplementary material for: The Perceived Roles of AI in Clinical Practice: National Survey of 941 Academic Physicians
Source: JMIR AI. 2025 Dec 4;4:e72535. doi: 10.2196/72535 (PMC12715463; doi:10.2196/72535)
Supplement: Multimedia Appendix 1 [file ai_v4i1e72535_app1.pdf]

AI in Medicine Survey

Q1 Please indicate your current position.

- ○ Attending
- ○ Resident
- ○ Other ____

Q2 Please indicate your specialty.

- ☐ Anesthesiology & Perioperative Medicine
- ☐ Dermatology
- ☐ Emergency Medicine
- ☐ Family Medicine
- ☐ Head and Neck Surgery
- ☐ Internal Medicine
- ☐ Neurology
- ☐ Neurosurgery
- ☐ Obstetrics and Gynecology
- ☐ Ophthalmology
- ☐ Orthopedic Surgery
- ☐ Pathology and Laboratory Medicine
- ☐ Pediatrics
- ☐ Plastic Surgery

☐ Psychiatry and Biobehavioral Sciences

☐ Radiation Oncology

☐ Radiology

☐ Surgery

☐ Urology

☐ Other (please specify): ____

Display this question:

If please indicate your specialty. = Internal Medicine

Q2A Please indicate your subspecialty.

○ Cardiovascular Medicine

○ Gastroenterology and Hepatology

○ General Medicine

○ Genetic Medicine

○ Geriatric and Palliative Medicine

○ Hospital Medicine

○ Infectious Disease

○ Metabolism, Endocrinology & Diabetes

○ Pulmonary and Critical Care

○ Rheumatology

○ Other ____

Display this question:

If please indicate your specialty. = Surgery

Q2B Please indicate your subspecialty.

- ○ Cardiac Surgery
- ○ General Surgery
- ○ Oral and Maxillofacial
- ○ Pediatric Surgery
- ○ Plastic Surgery
- ○ Thoracic Surgery
- ○ Transplant Surgery
- ○ Vascular Surgery
- ○ Other ____

Q3 How many years have you been in practice?

____

Q4 Please indicate your type(s) of practice.

- ▢ Hospital Owned Practice
- ▢ Physician Owned Practice
- ▢ Government Practice
- ▢ University Practice
- ▢ Other ____

Q4A What setting do you practice in?

○ Urban

○ Rural

○ Other ____

Q5 What state(s) do you currently practice in?

▢ Alabama

▢ Alaska

▢ Arizona

▢ Arkansas

▢ California

▢ Colorado

▢ Connecticut

▢ Delaware

▢ Florida

▢ Georgia

▢ Hawaii

▢ Idaho

▢ Illinois

▢ Indiana

- [ ] Iowa
- [ ] Kansas
- [ ] Kentucky
- [ ] Louisiana
- [ ] Maine
- [ ] Maryland
- [ ] Massachusetts
- [ ] Michigan
- [ ] Minnesota
- [ ] Mississippi
- [ ] Missouri
- [ ] Montana
- [ ] Nebraska
- [ ] Nevada
- [ ] New Hampshire
- [ ] New Jersey
- [ ] New Mexico
- [ ] New York

- ☐ North Carolina
- ☐ North Dakota
- ☐ Ohio
- ☐ Oklahoma
- ☐ Oregon
- ☐ Pennsylvania
- ☐ Rhode Island
- ☐ South Carolina
- ☐ South Dakota
- ☐ Tennessee
- ☐ Texas
- ☐ Utah
- ☐ Vermont
- ☐ Virginia
- ☐ Washington
- ☐ West Virginia
- ☐ Wisconsin
- ☐ Wyoming

☐ Other/Not Practicing in the US

Q6 Does your hospital/healthcare system use or allow for the use of AI in clinical practice?

◯ Yes, my healthcare system uses or allows for the use of AI in my specialty.

◯ No, my healthcare system does not use or allow for the use of AI in my specialty.

◯ I do not know.

Display this question:

If does your hospital/healthcare system use or allow for the use of AI in clinical practice? = Yes, my healthcare system uses or allows for the use of AI in my specialty.

Q6A Do you currently use artificial intelligence (AI) in clinical practice?

◯ Yes

◯ No

Display this question:

If does your hospital/healthcare system use or allow for the use of AI in clinical practice? = No, my healthcare system does not use or allow for the use of AI in my specialty.

Or does your hospital/healthcare system use or allow for the use of AI in clinical practice? = I do not know

Q6B Would you use AI in clinical practice if your healthcare system made it clinically accessible?

◯ Yes

◯ No

Display this question:

If do you currently use artificial intelligence (AI) in clinical practice? = Yes

Q7 If you selected yes, how do you use AI in practice? Please select all that apply.

- ☐ Image Analysis/Image Segmentation
- ☐ Surgical Decision Making
- ☐ Clinical Decision Making
- ☐ Risk Stratification
- ☐ Diagnosis
- ☐ Prognosis
- ☐ I do not use AI in practice.
- ☐ Other ____

Display this question:

If do you currently use artificial intelligence (AI) in clinical practice? = Yes

Q7A If you selected yes, why do you use AI? Please select all that apply.

- ☐ AI provides additional, objective metrics to consider in clinical practice.
- ☐ AI is useful for predicting patient/disease outcomes.
- ☐ AI is useful for data analysis in a time-efficient manner.
- ☐ AI is useful for reducing errors in practice and decision making.
- ☐ I do not use AI in practice (please go back)
- ☐ Other ____

Display this question:

If you would use AI in clinical practice if your healthcare system made it clinically accessible? = Yes

Q7B If you could use AI in clinical practice, how would you like to use it? Please select all that apply.

- [ ] Image Analysis/Image Segmentation
- [ ] Surgical Decision Making
- [ ] Clinical Decision Making
- [ ] Risk Stratification
- [ ] Diagnosis
- [ ] Prognosis
- [ ] Providing objective solutions
- [ ] Others ____

Display this question:

If do you currently use artificial intelligence (AI) in clinical practice? = No

Q8 If you selected no, why do you not use AI? Please select all that apply.

- ☐ I do not trust AI to provide accurate results to inform my decision making.
- ☐ There is no financial incentive for me to use it in clinic/surgery.
- ☐ I do not believe AI adds substantially to my clinical decision making.
- ☐ I do not feel the need to supplement my clinical judgement with AI.
- ☐ I have ethical concerns about using AI in practice.
- ☐ I do not understand AI well enough to use it in clinical practice.
- ☐ Legal concerns
- ☐ I do not know of any models that answer relevant clinical questions to my scope of practice (please indicate your specialty/subspecialty below). ____
- ☐ Other ____

Display this question:

If would you use AI in clinical practice if your healthcare system made it clinically accessible? = No

Q9 If you selected no, why would you not use AI? Please select all that apply.

- ☐ I do not trust AI to provide accurate results to inform my decision making.
- ☐ There is no financial incentive for me to use it in clinic/surgery.
- ☐ I do not believe AI adds substantially to my clinical decision making.
- ☐ I do not feel the need to supplement my clinical judgement with AI.
- ☐ I have ethical concerns about using AI in practice.
- ☐ I do not understand AI well enough to use it in clinical practice.
- ☐ Legal concerns
- ☐ I do not know of any models that answer relevant clinical questions to my scope of practice (please indicate your specialty/subspecialty below).
____
- ☐ Other ____

Q10 What would encourage you to increase your use of AI in clinical practice? Please select all that apply.

- ☐ Better integration into clinical workflow.
- ☐ Financial incentives (ex. CPT codes).
- ☐ Increased validation of current models.
- ☐ Proof of the utility/success of AI in current clinical practice.
- ☐ Other ____

Q11 What reservations or ethical concerns do you have about using AI in clinical practice? Please select all that apply.

- ☐ Clinical errors made by AI.
- ☐ Safety of patient data
- ☐ Concerns about AI replacing my clinical duties.
- ☐ AI replicating and perpetuating human bias.
- ☐ I do not have ethical concerns.
- ☐ Other ____
